# Transcriptomic and metabolic flux analyses reveal shift of metabolic patterns during rice grain development

**DOI:** 10.1186/s12918-018-0574-x

**Published:** 2018-04-24

**Authors:** Fangzhou Shen, Xueting Wu, Luoxi Shi, Hang Zhang, Yangmin Chen, Xiaoquan Qi, Zhuo Wang, Xuan Li

**Affiliations:** 1 Bio-X Institutes, Key laboratory for the Genetics of Developmental and Neuropsychiatric Disorders (Ministry of Education), Shanghai Jiao Tong University, Shanghai, 200030 People’s Republic of China; 2 Key Laboratory of Synthetic Biology, CAS Center for Excellence in Molecular Plant Sciences, Institute of Plant Physiology and Ecology, Shanghai Institutes for Biological Sciences, Chinese Academy of Sciences, Shanghai, 200032 People’s Republic of China; 3 School of Life Sciences and Biotechnology, Shanghai Jiao Tong University, Shanghai, 200240 People’s Republic of China; 4 Division of Biostatistics, School of Public Health, University of Minnesota, Minneapolis, 55455 USA; 5 The Key Laboratory of Plant Molecular Physiology, Institute of Botany, Chinese Academy of Sciences, Beijing, 100093 People’s Republic of China

**Keywords:** Transcriptome, Metabolic flux, Tissue-specific model, Rice development, Secondary metabolism

## Abstract

**Background:**

Rice (*Oryza sativa*) is one of the most important grain crops, which serves as food source for nearly half of the world population. The study of rice development process as well as related strategies for production has made significant progress. However, the comprehensive study on development of different rice tissues at both transcriptomic and metabolic flux level across different stages was lacked.

**Results:**

In this study, we performed RNA-Seq and characterized the expression profiles of differentiated tissues from *Oryza sativa* Zhonghua 11, including leaves, sheath, stamen, pistil, lemma and palea of the booting stage, and embryo, endosperm, lemma and palea of the mature grain stage. By integrating this set of transcriptome data of different rice tissues at different stages with a genome-scale rice metabolic model, we generated tissue-specific models and investigated the shift of metabolic patterns, and the discrepancy between transcriptomic and metabolic level. We found although the flux patterns are not very similar with the gene expression pattern, the tissues at booting stage and mature grain stage can be separately clustered by primary metabolism at either level. While the gene expression and flux distribution of secondary metabolism is more diverse across tissues and stages. The critical rate-limiting reactions and pathways were also identified. In addition, we compared the patterns of the same tissue at different stages and the different tissues at same stage. There are more altered pathways at gene expression level than metabolic level, which indicate the metabolism is more robust to reflect the phenotype, and might largely because of the complex post-transcriptional modification.

**Conclusions:**

The tissue-specific models revealed more detail metabolic pattern shift among different tissues and stages, which is of great significance to uncover mechanism of rice grain development and further improve production and quality of rice.

**Electronic supplementary material:**

The online version of this article (10.1186/s12918-018-0574-x) contains supplementary material, which is available to authorized users.

## Background

Rice (*Oryza sativa*) is one of the most important grain crops in the world, which serves as food source for nearly half of the world population. Rice is also a model organism for research in plant growth and development. The process of rice growth and development starts from seed germination and ends when the formation of the grain begins. This complex process can be divided into three periods: vegetative growth, reproductive growth, grain filling and maturity. These three main phases correspond to the development of different tissues and organs in rice. Since it is important for the agricultural production of rice, the study of rice development process as well as related strategies for production has made significant progress [1, 2]. However the continuous analysis across different development stages by high-throughput technology was relatively limited. Sharma et al. categorized panicle and seed development into nine and five categories respectively, and used Affymetrix arrays to generate spatial and temporal expression profiles during rice reproductive organ development [3]. The dataset of differentially expressed genes was further filtered to identify genes that expressed specifically in one or more stages of panicle and seed development. Han et al. performed a gel-based comparative phosphoproteomic study on rice embryo during the germination process, and found phosphorylation of signal transduction proteins was mainly activated during the early stage of germination, while stress response and storage protein phosphorylation were enhanced at the late stage [4]. Gao et al. used RNA-Seq technique to reveal the molecular mechanisms involved in rice endosperm development [1].

It is well known that metabolism is a representation of the phenotype [5, 6], but few studies have been conducted on metabolic level of rice development. Recently, the dynamic metabolic changes along the rice grain development of two japonica and two indica cultivars were investigated using non-targeted metabolomics and 214 key metabolites were identified [7]. However, the comprehensive study on development of different rice tissues at both transcriptomic and metabolic flux level across different stages was lacked. Constraint-based metabolic models employ stoichiometric, thermodynamic, flux capacity and possibly other constraints to determine the space of possible flux distributions attainable by the network. Flux balance analysis (FBA) is a commonly used constraint-based approach that assumes the organism maximizes its biomass production rate [8], which has been used successfully to predict various metabolic phenotypes [9–12]. With the advancement of high-throughput omics technology, approaches have been developed to integrate metabolic model with gene expression data to improve the prediction of metabolic states. For instance, using Gene Inactivity Moderated by Metabolism and Expression (GIMME) method, the context-specific networks for *E. coli* have been constructed for different types of strains with different carbon source [13]. For Yeast grown on YPD (yeast-extract, peptone, dextrose) or YPEtOH (yeast-extract, peptone, ethanol), the environment-specific models have been built based on the Exploration of Alternative Metabolic Optima (EXAMO) method [14]. Another method called E-Flux extends flux bounds by converting measured gene expression using suitable function, which was applied to Mycobacterium tuberculosis [15]. For human, tissue-specific models by the integrative Metabolic Analysis Tool (iMAT) have been used to predict the tissue-specific metabolism and disease marker [16]. In addition, Agren et al. used INIT (Integrative Network Inference for Tissues) to reconstruct specific cancer models [17]. To date, very few studies integrate gene expression profiles with metabolic model in plants. Here we selected iMAT approach for construction of rice specific models. iMAT predicts the metabolic flux distribution by maximizing the number of enzymes whose predicted flux activity is consistent with their measured expression level, which does not require an objective function to be predefined, such as biomass or particular product. Another advantage of iMAT is that the post-transcriptional regulation could be identified by checking the inconsistencies between gene expression and metabolic flux.

**Fig. 1:**
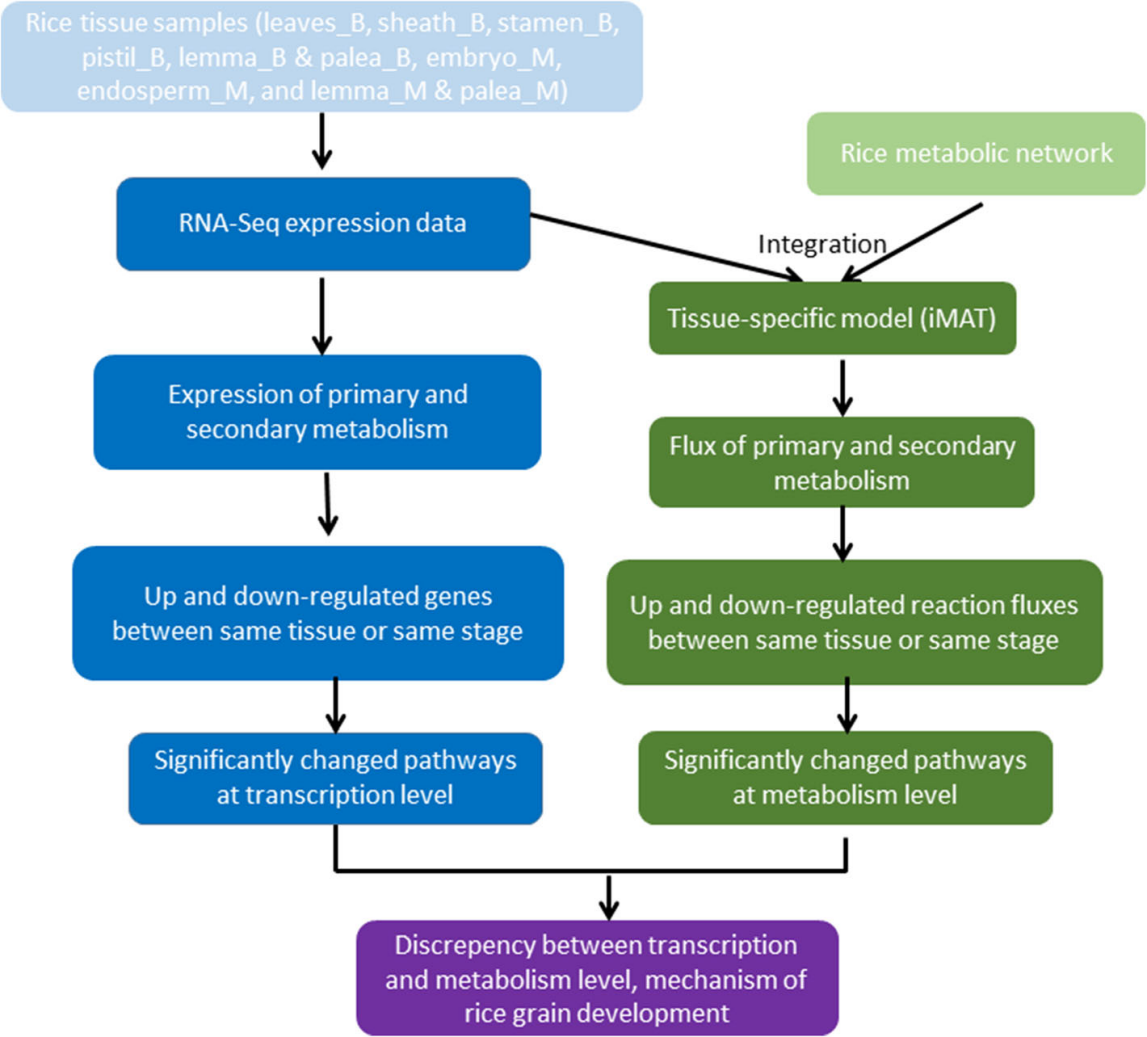
The design and analysis pipeline of metabolic pattern shift across different rice tissues during different stages

In this study, by integrating transcriptome data of different rice tissues with a genome-scale rice metabolic model, we generated tissue-specific models and investigated the shift of metabolic patterns, and the discrepancy between transcriptomic and metabolic level. We found although the flux patterns are not very similar with the gene expression pattern, the tissues at booting stage and mature grain stage can be separately clustered by primary metabolism at either level. While the gene expression and flux distribution of secondary metabolism is more diverse across tissues and stages. The critical rate-limiting reactions and pathways were also identified. We compared the pairs of samples of the same tissues at different stages or the different tissues at same stage to uncover the difference between transcription level and metabolism level. Three compared pairs showed there are more altered pathways at gene expression level than metabolic level, which indicated metabolism is more robust to reflect the phenotype. The analysis pipeline of this study is shown in Fig. 1. In conclusion, the metabolic model combined with transcriptome data pipeline revealed more detail metabolic pattern shift among different tissues and stages, which can be exploited to uncover the mechanism of the complex metabolic behavior of rice and further improve production and quality of rice.

## Results

### Transcriptomic profiles of stage-specific rice tissues

**Fig. 2:**
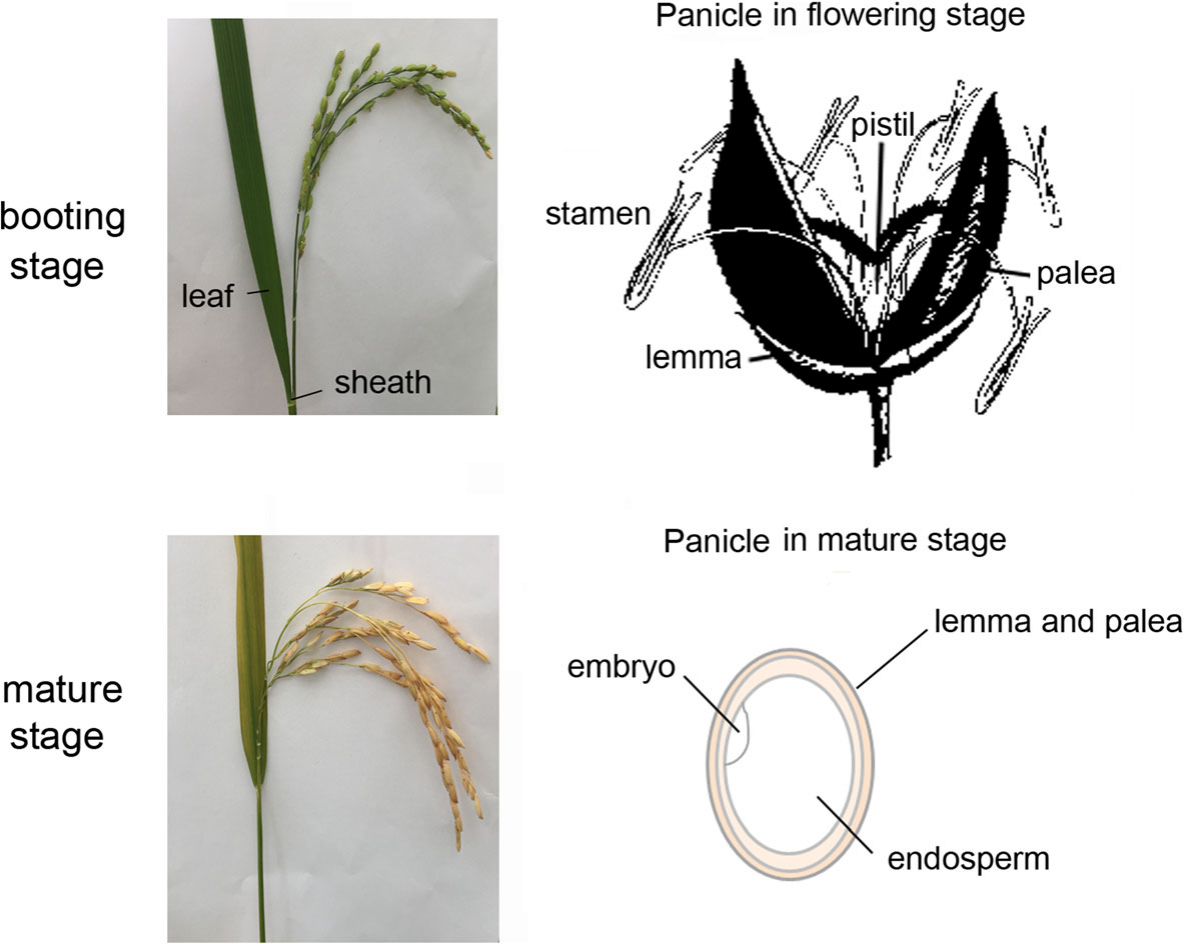
Materials of Oryza.sativa collected in the study. Leaves, sheath, stamen, pistil, lemma and palea of booting stage, embryo, endosperm and lemma and palea of mature stage were collected respectively. Panicle of rice in the flowering stage can be enlarged to exhibit stamen, pistil, lemma and palea of booting stage, while panicle of rice in the mature stage can be enlarged to exhibit the seed which composed of embryo, endosperm and lemma and palea of mature stage

To achieve and characterize the expression profiles of *Oryza sativa*, deep sequencing on RNA of differentiated tissues from *Oryza sativa* was performed. The tissue samples of Zhonghua 11 were collected from leaves, sheath, stamen, pistil, lemma and palea of the booting stage (leaves_B, sheath_B, stamen_B, pistil_B, lemma_B & palea_B), and from embryo, endosperm, and lemma and palea of the mature grain stage (embryo_M, endosperm_M, and lemma_M & palea_M, Fig. 2), from which total RNA was isolated as described in Methods. The RNA-Seq libraries were sequenced using Illumina HiSeq2500 platform, with a paired-end read length of 125 base pairs (bp). A total of 318 million reads (approximate 40 Gbp) were obtained for all eight *Oryza sativa* tissues (Table 1). Then low-quality reads, and adaptor or ambiguous sequences were filtered according to fastq_quality_filter of fastx_toolkit-0.0.14(http://hannonlab.cshl.edu/fastx_toolkit/index.html), high-quality clean reads were retained for subsequent analyses.

**Table 1:** Overview of the sequencing and mapping of transcriptome data of different rice tissues

Stage	Tissue	RawData(bp)	RawData (number)	CleanData (number)	Mapped reads(number)	Mapping rate
Booting stage	leaves	4,392,290,250	35,138,322	26,531,310	24,899,774	93.9%
	sheath	5,873,023,000	46,984,184	36,659,446	34,301,867	93.6%
	stamen	6,059,084,500	48,472,676	38,746,796	36,425,576	94.0%
	pistil	4,348,780,750	34,790,246	26,154,367	24,861,601	95.1%
	lemma & palea	4,673,388,000	37,387,104	30,018,313	28,174,883	93.9%
Mature grain stage	embryo	4,524,559,250	36,196,474	30,212,929	27,930,200	92.4%
	endosperm	4,892,736,750	39,141,894	27,017,181	25,101,827	92.9%
	lemma & palea	5,028,140,000	40,225,120	29,610,747	27,684,908	93.5%

**Fig. 3:**
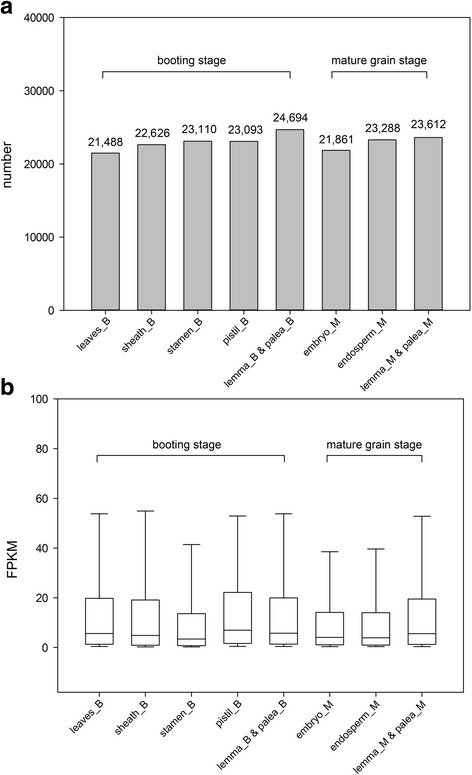
Transcripts expressed number and level in different tissues. **a** The number of transcripts expressed (FPKM> 0) in each tissue was labeled over the bars. **b** Expression levels of transcripts in the eight tissues

To analyze the transcriptomic profile of *Oryza sativa* and its gene models, reads were filtered from the eight tissues and then mapped separately to the *Oryza sativa* reference genome (downloaded from http://rapdb.dna.affrc.go.jp/download/irgsp1.html). About 229,380,636 (~ 72%, Table 1) filtered reads (paired-end and directional) were mapped to the *Oryza sativa* genome using TopHat (version 2.0.12) [18] with default parameters. Gene models were reconstructed by Cufflinks (version 2.2.1) [19] based on the alignment results from TopHat, the numbers of genes in each tissue was labeled in Fig. 3a. Gene expression levels were estimated by computing the FPKM value (Fragment per Kilobase per Million mapped reads) for transcripts of each tissue (Fig. 3b) [19].

### Differential expression pattern of genes in primary and secondary metabolic processes

**Fig. 4:**
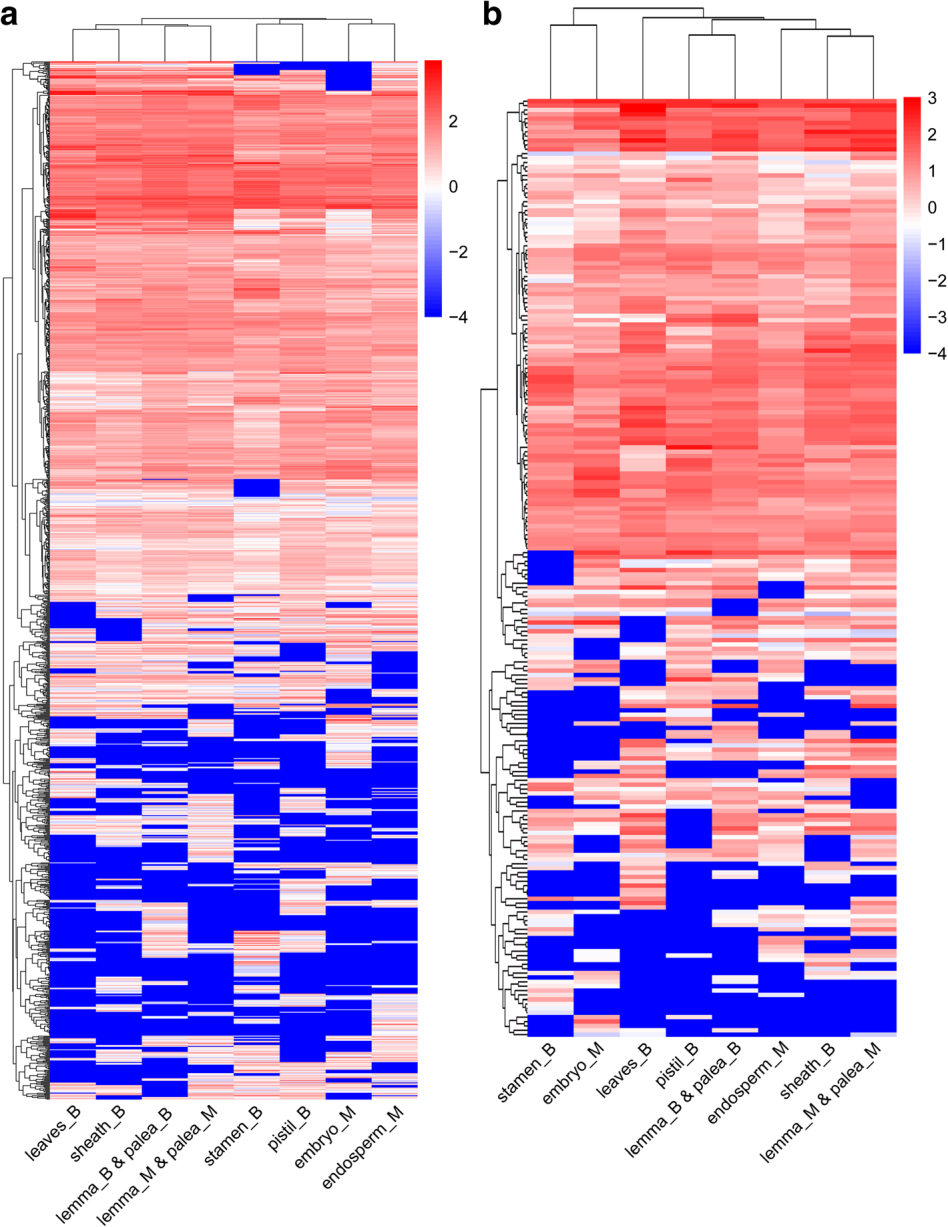
Heatmap of gene expression in primary and secondary metabolic processes. **a** Heatmap of gene expression(*log*_10_(*fpkm*)) in primary metabolic processes. **b** Heatmap of gene expression(*log*_10_(*fpkm*)) in secondary metabolic processes

Apart from producing primary metabolites which are essential to the growth of the cell and involved directly in metabolic reactions, plants also synthesize a myriad of secondary metabolites that are derived from central or primary metabolism [20, 21]. Secondary metabolites are the compounds which are not essential to sustain the life of cells, most of which are involved in defense reactions. As there are many differences between primary and secondary metabolism, we aimed to find the difference between gene expression patterns of *Oryza sativa* in primary and secondary metabolic processes. There are 1216 and 214 genes respectively mapped to the primary and secondary metabolic processes, which were shown in Additional file 1. As shown in Fig. 4, genes in primary and secondary metabolic processes revealed different expression pattern among eight tissues of *Oryza sativa*. In the primary processes, genes of lemma and palea of two different stages, sheath_B and leaves_B, pistil_B and stamen_B, endosperm_M and embryo_M clustered together, respectively (Fig. 4a), which demonstrated the tissues with correlated function have similar expression profile of primary metabolism. While for the secondary metabolic processes, gene expression of different tissues clustered more randomly (Fig. 4b), which means the gene expression of secondary metabolism are more diverse across tissues and stages. This may be on account of certain secondary metabolic responses only occur in a particular species, organs, or tissues under certain environmental and temporal conditions.

### Construction and analysis of tissue-specific models at booting and mature grain stages

**Fig. 5:**
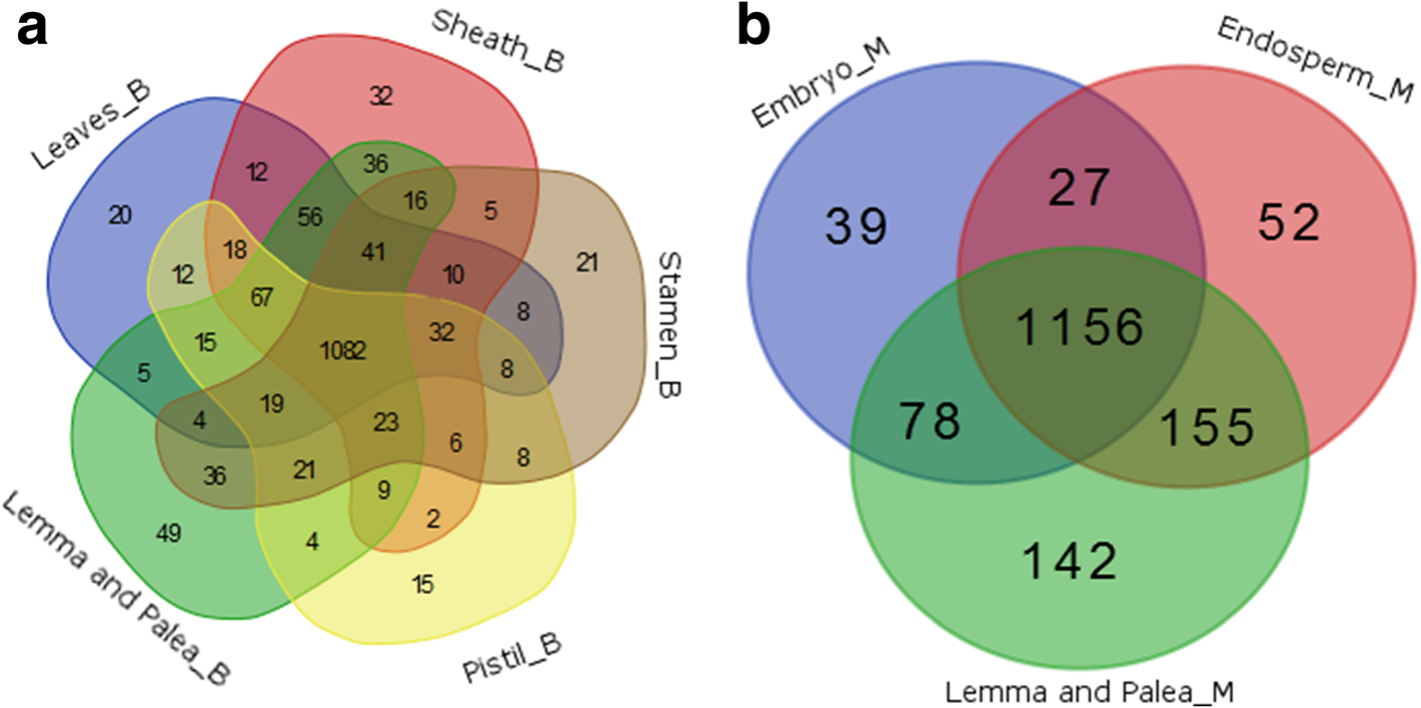
Venn Diagrams of the number of active reactions in tissue-specific models in booting stage (**a**) and mature grain stage (**b**)

We integrated transcriptome data with the metabolic model of rice (iOS2164) [22] and constructed tissue-specific models of rice by using the Integrative Metabolic Analysis Tool (iMAT)method [23]. The principle of iMAT is maximizing the number of reactions whose activity is consistent with their expression state by formulating a mixed integer linear programming (MILP) problem, and then the specific model will be generated including only active reactions. The detail is described in Methods section and the input data of iMAT is in Additional file 2. Figure 5 showed the number of reactions in different tissue-specific models in booting stage and mature grain stage. There are average half of the total 2445 reactions active in the specific models, among which 1082 and 1156 reactions are shared respectively by all tissues in booting stage and mature grain stage. We found more reactions are specifically required in palea and lemma in both of the two stages, which indicated that more reactions are required for cell growth and development in palea and lemma. This might probably because compared with the other organs, palea and lemma are more resistant to drought stress and might be major sources of carbon in grain-filling stage under drought, which might require more reactions to support cell growth and development [24].

**Fig. 6:**
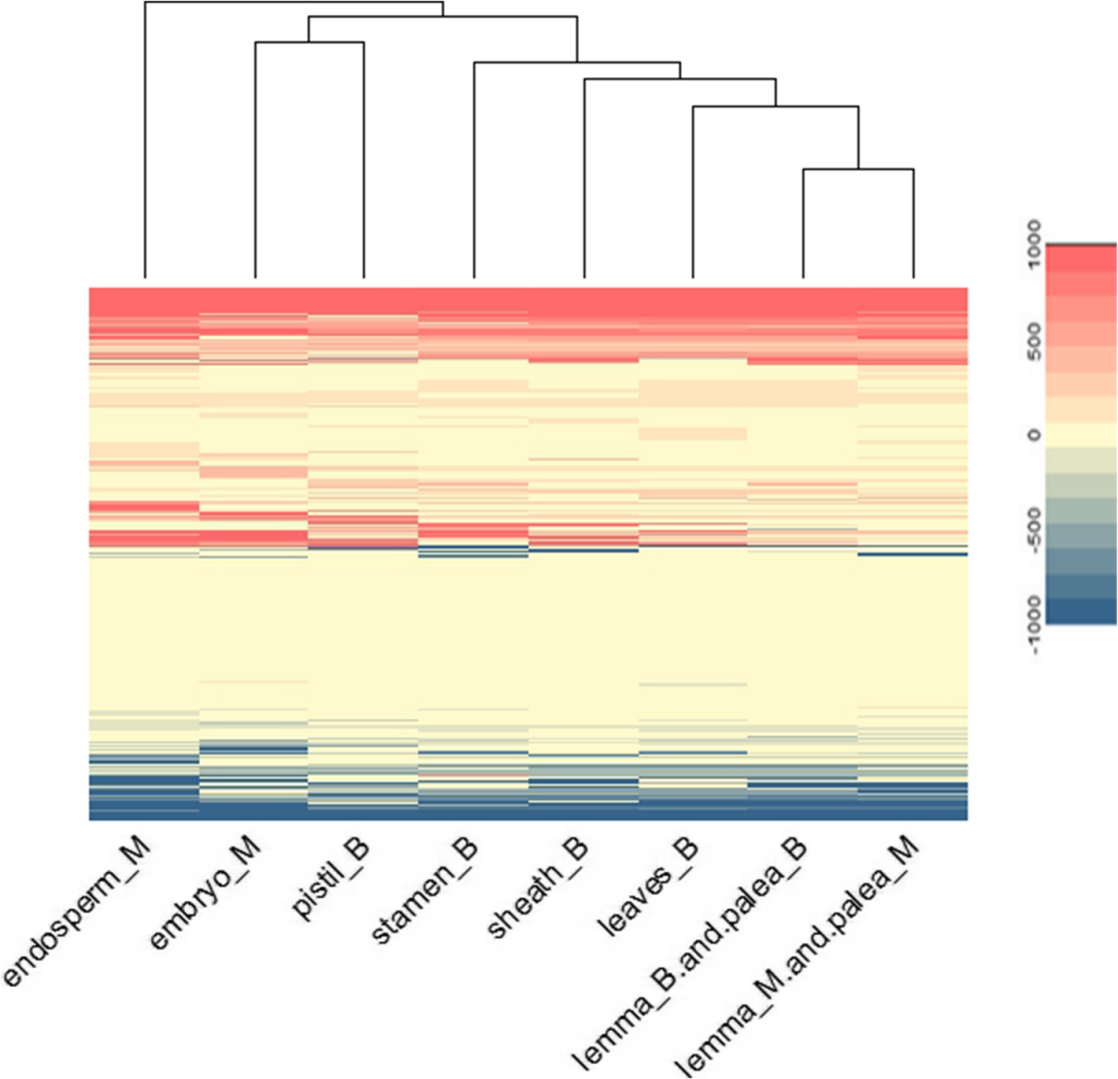
Metabolic flux of tissues in booting and mature grain stage

We simulated the metabolic flux of all the tissue-specific models, the range of flux value is [− 1000,1000], where negative number means reverse reaction flux. We directly used the flux value to measure the pattern similarity and made hierarchical clustering as shown in Fig. 6. Leaf, sheath, palea and lemma in booting stage have similar flux distribution. There are more specific active reactions in leaves and pistil in booting stage, as well as embryo and endosperm in mature stage. There are more inactive reactions specifically in leaves in booting stage and embryo in maturity stage.

By evaluating the total variation (see Methods) of every reaction across the 8 different stages and tissues, we found some pathways exhibit significant changes, including glycolysis/gluconeogenesis, fatty acid metabolism, nitrogen metabolism, TCA cycle, glyoxylate cycle, cysteine and methionine metabolism, photosynthesis (light phosphorylation), folate metabolism, and fermentation process.

### Metabolic flux of primary metabolism across different tissues and stages

**Fig. 7:**
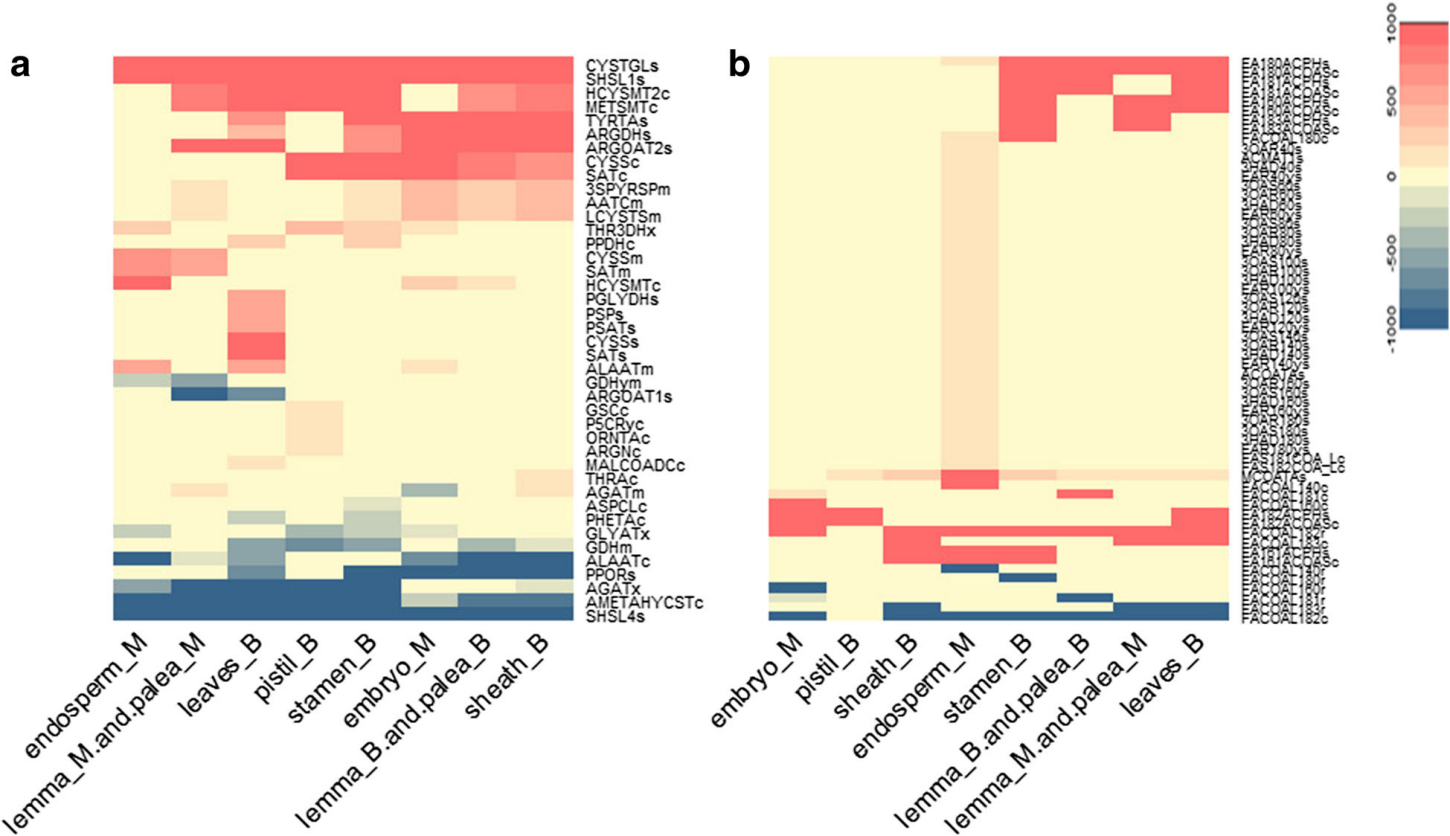
Flux distribution of fatty acid metabolism (**a**) and amino acid metabolism (**b**)

The flux distribution of primary metabolism in 8 tissue-specific models were shown in Additional file 3, lemma and palea of two different stages have similar flux pattern, then together with leaves_B, sheath_B and stamen_B to make one cluster, while endosperm_M, embryo_M and pistil_B form another cluster. Although the flux pattern are not very similar with the gene expression pattern, the tissues in booting stage and mature grain stage can be separately clustered by primary metabolism at either level. We investigated the enriched pathways of significantly active reactions. In all tissue-specific models, Glycine, serine and threonine metabolism are significantly active (Fig. 7). Calvin cycle is significantly active in mature lemma and palea. Glycerolipid and Glycerophospholipid metabolism are significantly active in embryo of mature stage. Phenylalanine, tyrosine and tryptophan metabolism is significantly active in pistil of booting stage. Purine metabolism is significantly active in both leaves and pistil of booting stage. Pyrimidine metabolism is significantly active in both leaves and stamen of booting stage.

We determined the reactions achieving upper bound of flux as rate-limiting steps, and then selected the pathways enriched in the rate-limiting reactions. Glycolysis/Gluconeogensis pathway is found to be rate limited in all tissue-specific models, Fatty acid, Porphyrin and Chlorophyll Metabolism are also rate limited in most of the tissues. These observations are in good agreement with the point that the basal metabolism maintains the most basic energy expenditure of the organism’s life activities. In comparison, folate metabolism and TCA cycle are rate-limiting steps in only two or three tissues. Nitrogen metabolism is rate-limiting only in leaves at booting stage, and glyoxylate cycle is rate-limiting step only in endosperm at mature stage. It is consistent with previous studies that ${NO}_2^{-}$ assimilation takes place in chloroplast, and Nitrogen metabolism requires organic carbon and energy from CO2 assimilation and photosynthesis [25]. The glyoxylate cycle plays an important role in the growth and development of plants, which turns fat that relatively abundant in endosperm into sugar [26]. Moreover, these pathways are highly fluctuant across different tissues at different stages, which indicate that these pathways are more important specifically in particular tissues.

Next, we focused on the metabolic features of some fundamental processes, the flux distribution of Fatty acid metabolism and Amino acid metabolism are shown in Fig. 7. The activity of fatty acid metabolism in pistil at booting stage is lower than in other tissues and development stages. The elongation of fatty acid exhibits divergence because the specific reactions in different tissues correspond to the elongation of fatty acid with different length. There are 35 specifically activated reactions in endosperm, so we concluded that endosperm is more active in fatty acid metabolism than other tissues, which is beneficial for nutrient accumulation in endosperm. We found that synthesis of serine is specifically active in leaves at booting stage.

**Fig. 8:**
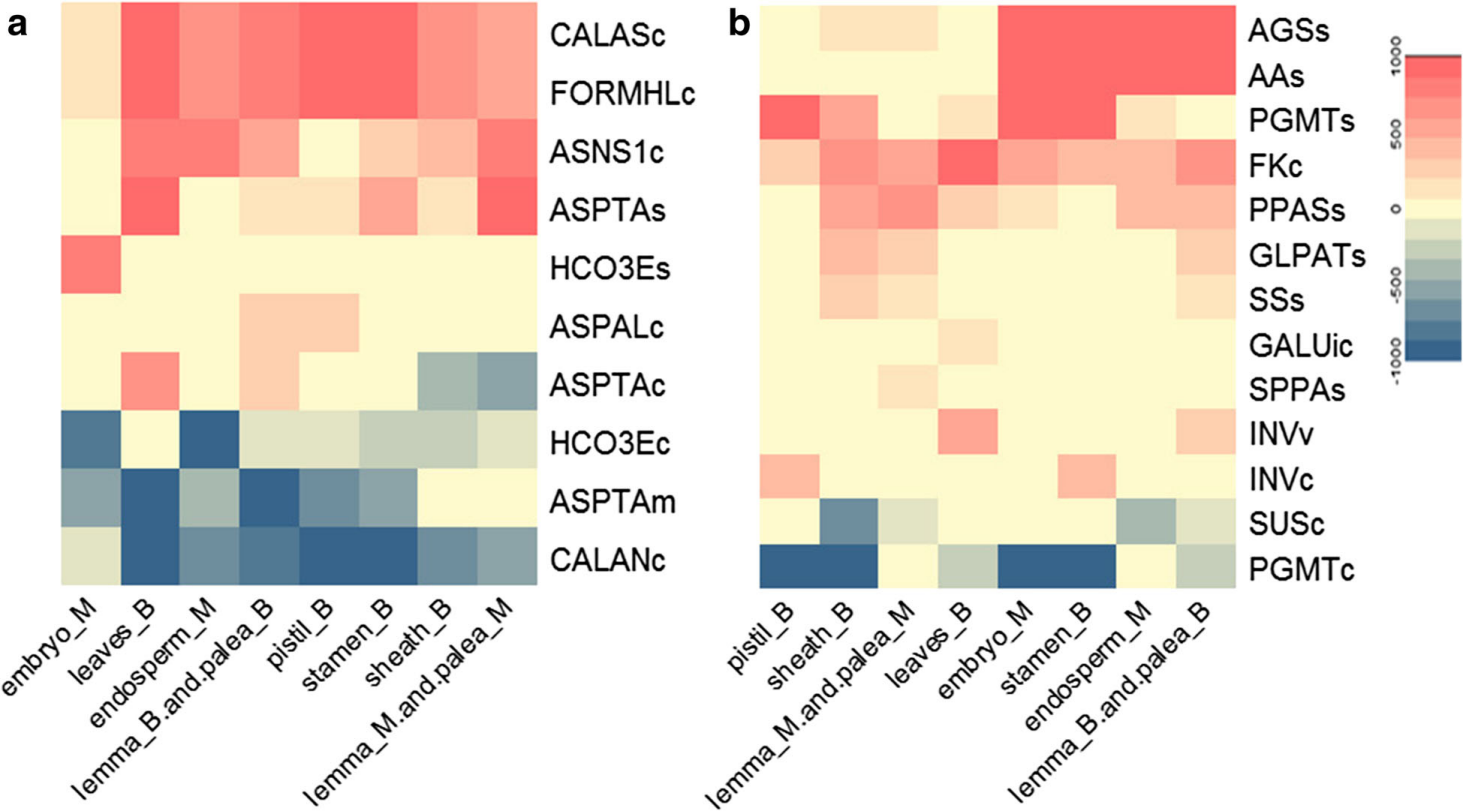
Flux distribution of nitrogen metabolism(**a**) and starch and sucrose metabolism (**b**)

The flux distribution of Nitrogen metabolism and Starch and sucrose metabolism are shown in Fig. 8. In embryo at maturity stage, the activity of nitrogen metabolism is significantly lower than other tissues. Stamen, palea and lemma in booting stage are more active in starch metabolism. And there are no significant similarities of sucrose metabolism in different tissues.

### Metabolic flux changes in secondary metabolism among different tissues

**Fig. 9:**
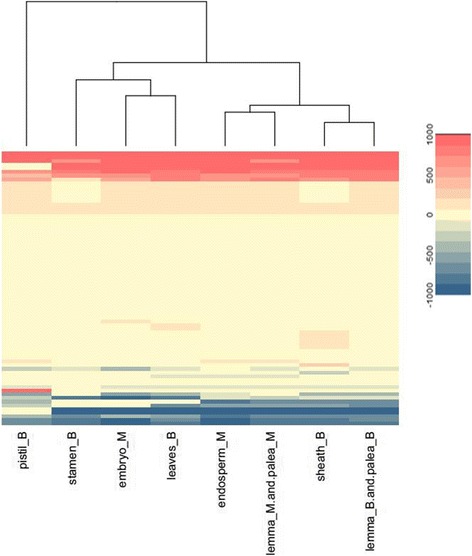
Metabolic flux distribution of secondary metabolism in different tissues at booting and mature grain stages

The secondary metabolism of plants has very important effect on orchestrating the cellular phenotype in response to abiotic stresses [27, 28]. In this study, the comprehensive design and measurements of gene expression for 8 tissues at booting and mature grain stages enabled the systematic analysis of secondary metabolism for rice development. We classified the reactions in secondary metabolic pathways according to annotation in rice model (Additional file 1), and plotted the flux heatmap of these reactions as shown in Fig. 9, the clusters did not distinguish in terms of stages or correlated tissues, which means flux pattern of secondary metabolism is also divergent as the gene expression level. We found the pathways of biotin, Vitamin E, abscisic, ethylene, brassinosteriods, B6, jasmonic, zeatin, and gibberallins are significantly inactive in all tissues at both booting and mature grain stages. On the contrary, Riboflavin Metabolism and abscisic acid biosynthesis are significantly active in all tissues. As known to all, riboflavin involves in the induction of defense responses in human, animals, plants, and microorganisms, by interfering in antioxidation [29], peroxidation [30], or activation of several defense mechanisms [31]. Riboflavin is referred as an effective plant defense activator against different fungal, bacterial, and viral pathogens when applied exogenously on dicot plants such as Arabidopsis and tobacco [32]. The activity of riboflavin metabolism in all tissues to some extent indicated that it also acts as a defense activator in *Oryza sativa* (monocot plant) [32]. Abscisic acid is one of the plant hormones that acted as an important signal molecule for abiotic stress adaptation, and also acts as a developmental signal [33].

Flux distributions in quinone and folates metabolism are similar in all tissues. Folates (vitamin B9) and phylloquinone (vitamin K1) have potential antioxidant activity within the plant cell [34]. Stamen and pistil have similar flux distributions in terpenoid and flavonoid metabolism. Terpenoids are involved in diverse biological processes ranging from plant defense to reproduction and symbiosis [35], by synthesized as the components of resins, complex oils, or volatile mixtures (such as floral scents) [35, 36]. Stamen and sheath have similar flux distributions in phenylpropanoid. Systemic accumulation of phenylpropanoids in rice can enhance capabilities for growth and development [37]. Embryo and endosperm have similar distributions in thiamine metabolism. The most divergent tissue in secondary metabolism is pistil with nicotinate specifically active, and porphyrin and chlorophyll specifically inactive.

### The comparison between gene expression and metabolic flux in same tissue or same stage

**Fig. 10:**
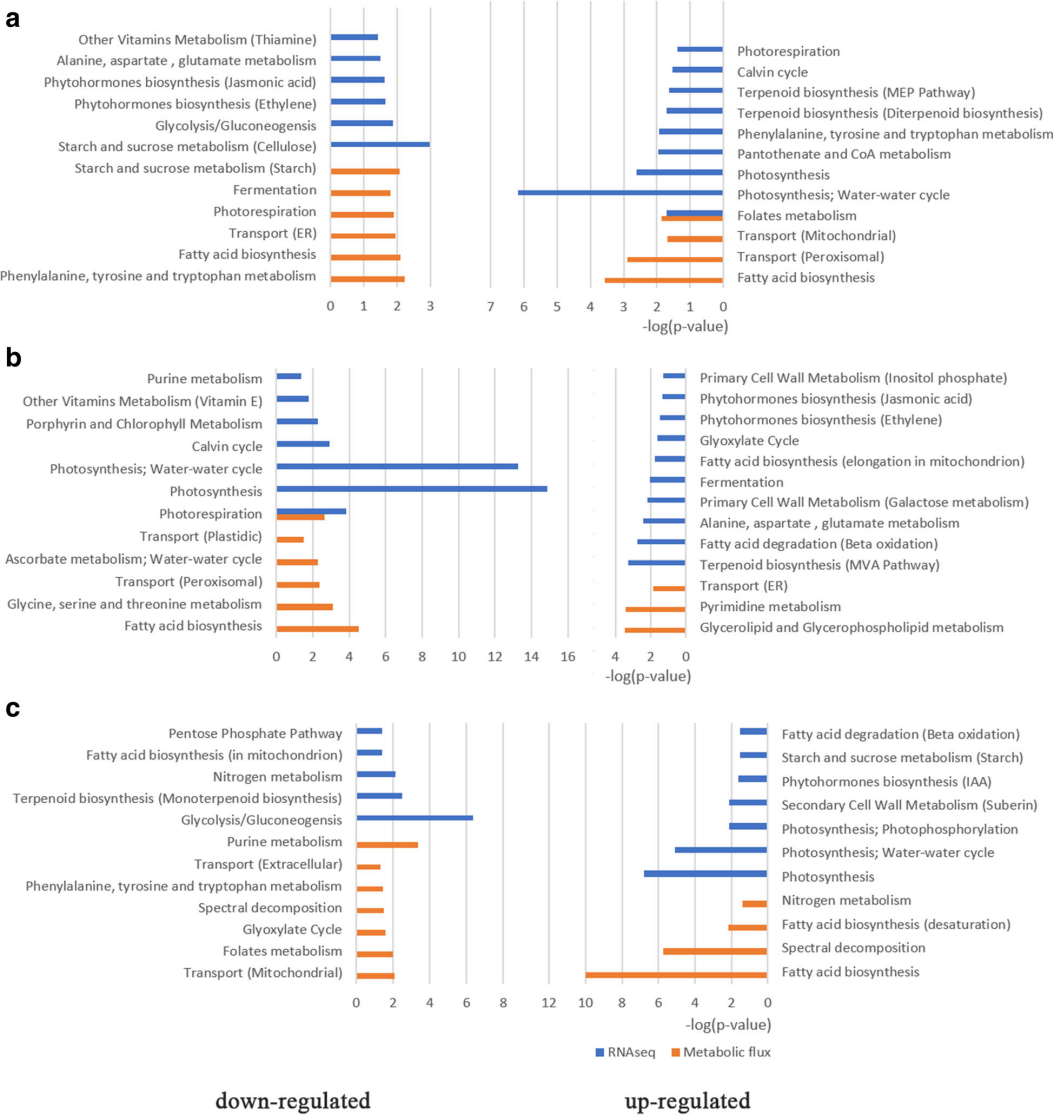
Pathway enrichment of down-regulated and up-regulated gene expression and metabolic flux in compared pairs of tissue models. **a** Palea and lemma in mature grain stage compared with booting stage. **b** Sheath compared with leaves in booting stage. **c** Endosperm compared with embryo in mature grain stage

There is a significant difference between the gene expression level and metabolic flux distribution, which indicate the importance of post-transcriptional control in organisms. And the integration of transcriptome data with metabolic model is important to analyze the metabolic changes. Here we compared the pairs of samples of the same tissues at different stages or the different tissues at same stage to uncover the difference between transcriptomic level and metabolic level. The significantly up- and down-regulated genes were determined by the fold change of fpkm larger than 2 or smaller than 0.5. While for the significantly up- and down-regulated reaction fluxes, we calculated the difference of absolute of two flux values and divided by the range of flux variability (See Methods). If the ratio is larger than 0.5, the reaction is upregulated; If the ratio is smaller than − 0.5, the reaction is downregulated. Then we conducted functional enrichment analysis to show the significantly up- and down-regulated pathways between three compared pairs at the two different levels in Fig. 10. The first pair is the palea and lemma at booting and mature stage, Fig. 10a demonstrated that starch metabolism, fermentation, photorespiration, fatty acid biosynthesis, and phenylalanine, tyrosine and tryptophan metabolism are downregulated in palea and lemma at mature stage, only folate and part of fatty acid biosynthesis are upregulated, therefore we concluded that palea and lemma develop mainly in booting stage. During the booting stage, the palea and lemma interlock with each other to enclose the developing floral organs [24, 38]. There are significant differences between transcriptomic and metabolic level, although gene expression of calvin cycle, photosynthesis, terpenoid metabolism are upregulated in mature palea and lemma, these processes have no significant changes in metabolic flux. While genes in photorespiration, phenylalanine, tyrosine and tryptophan metabolism were upregulated, the fluxes were downregulated. Thiamine and phytohormones pathways enriched in downregulated genes, but no significant changes in flux. This is largely because of the complex post-transcriptional modification [39, 40].

The second pair is the sheath and leaves both at booting stage. We found the process of photosynthesis and fatty acid biosynthesis are much stronger in leaves than in sheath, as shown in Fig. 10b, which is consistent with the actual situation that leaves are responsible for photosynthesis. Also there are more altered pathways at gene expression level than metabolic level, which indicate the metabolism is more robust to reflect the phenotype.

The third pair is embryo and endosperm both at mature grain stage. Rice is a representative of a monocotyledonous plant, which possesses a tiny embryo and large endosperm used for starch storage. During germination, sucrose has the potential to transport carbohydrate from endosperm to embryo. The endosperm of rice is a major storage tissue for starch and proteins, which is essential for embryo growth and postembryonic development prior to the start of seedling photosynthesis [41]. Previous studies have revealed endosperm development during early, middle and late stages by RNA-Seq [1], but the metabolic flux of reactions in endosperm is less studied. From Fig. 10c, we can see the starch and sucrose metabolism were upregulated in endosperm compared with embryo, which means endosperm can storage more starch [42]. The gene expression of nitrogen metabolism and fatty acid biosynthesis were downregulated in endosperm, but their fluxes were increased. Endosperm can accumulate more nutrients than embryo, and embryo may convert fatty acid into carbohydrate used in growth and development with glyoxylate cycle.

## Discussion

### Integration of transcriptome data with metabolic model is important to analyze rice development of different tissues

Transcriptome can reveal the gene expression level at different development stage, but in reality, the flow of information deriving from genes is networked by many loops with their downstream products, resulting in a complex and dynamic system of transcripts, proteins and metabolites. Therefore metabolic flux analysis can be applied to reveal the mechanism of rice development. By integration of gene expression data with the genome-scale rice metabolic model, we constructed tissue-specific models, and simulated the flux distribution, which can reflect the changes of metabolic features without further experimental measurements. There are different numbers of reactions in the 8 tissue-specific models, majority of them are common, but each has specific reactions. More reactions are specifically required in palea and lemma in both booting and mature grain stage for cell growth and development. This might probably because compared with other organs, the palea and lemma is more resistant to drought stress. Meanwhile, the palea and lemma may be the major sources of carbon for grain-filling during drought, which requires more reactions to support cell growth and development [24]. In addition, we identified the significantly altered pathways by total flux variation analyses, which include glycolysis/gluconeogenesis, fatty acid metabolism, nitrogen metabolism, TCA cycle, glyoxylate cycle, cysteine and methionine metabolism, photosynthesis (light phosphorylation), folate metabolism, and fermentation process. In comparison, other pathways exhibit similar flux distribution across 8 tissues. The construction of such plant metabolic models combined with transcriptome data could be challenging, mainly attributable to the physiological differences among the eight tissues, the localization of reactions, and the annotation of unidentified genomic functions [27, 43].

### Gene expression and metabolic flux of secondary metabolism are more diverse than primary metabolism among different tissues

Primary and secondary metabolism played different roles in rice development, and we revealed their different features at both transcriptomic and metabolic level. The transcriptome data indicated the gene expression of the primary processes could be categorized into four clusters: sheath and leaves, pistil and stamen, endosperm and embryo, lemma and palea of booting and mature grain stages (Fig. 3a), which demonstrated the tissues with correlated function have similar expression profile of primary metabolism. The clusters by flux pattern of primary metabolism also revealed that tissues at booting stage and mature grain stage can be separately classified. While for the secondary metabolic processes, different tissues clustered more randomly (Fig. 4b, Fig. 9), which means the gene expression and flux distribution of secondary metabolism are more diverse across tissues and stages. This may be due to that some specific secondary metabolic responses only happen in a particular species, organs, or tissues under certain environmental and temporal conditions. The rate limiting reactions are all involved in primary metabolic processes. Glycolysis/Gluconeogensis pathway is found to be rate limiting in all tissue-specific models, Fatty acid, Porphyrin and Chlorophyll Metabolism are also rate limiting in most of the tissues. These might because basal metabolism maintains the most basic energy expenditure of the organism’s life activities. Nitrogen metabolism is rate-limiting only in leaves at booting stage, because nitrogen is important for photosynthesis. ${NO}_2^{-}$assimilation occurs in chloroplast, Nitrogen metabolism requires organic carbon and energy from CO_2_ assimilation and photosynthesis as well as other electron transport chains [25]. And glyoxylate cycle is rate-limiting step only in endosperm at maturity stage. The glyoxylate cycle is essential for the growth and development of plants, which translates fat into sugar in endosperm [26]. The pathways of Biotin, Vitamin E, Abscisic, Ethylene, Brassinosteriods, B6, Jasmonic, Zeatin, and Gibberallins are significantly inactive in all tissues at both booting and maturity stages, which are not required for rice development. On the contrary, Riboflavin Metabolism and Abscisic acid biosynthesis are significantly active in all tissues. The activity of Riboflavin Metabolism indicates that it acts as a defense activator in *Oryza sativa* (monocot plant) [32]. Abscisic acid is one of the plant hormones that acts as an important signal molecule for abiotic stress adaptation, and also acts as a developmental signal [33]. Different tissues have different flux distribution in other secondary pathways, such as terpenoid, flavonoid, phenylpropanoids and thiamine metabolism. The most divergent tissue in secondary metabolism is pistil with Nicotinate specifically active, and Porphyrin and chlorophyll specifically inactive. The final determination of actual flux distributions among different tissues must await experimental verification through isotope-based internal flux measurements in the future.

### Difference between gene expression and metabolic flux revealed potential post-transcriptional modification

We compared the pairs of samples of the same tissues at different stages or the different tissues at same stage to uncover the difference between transcriptomic level and metabolic level. Three compared pairs showed there are more altered pathways at gene expression level than metabolic level, which indicate the metabolism is more robust to reflect the phenotype. By comparing palea and lemma at maturity stage with booting stage, we found although gene expression of calvin cycle, photosynthesis, terpenoid metabolism are upregulated in mature palea and lemma, these processes have no significant changes in metabolic flux. While genes in photorespiration, phenylalanine, tyrosine and tryptophan metabolism are upregulated, the fluxes are downregulated. Thiamine and phytohormones pathways are enriched in downregulated genes, but no significant changes in flux. Since more reactions including starch metabolism, fermentation, photorespiration, fatty acid biosynthesis, are downregulated in mature grain stage, we concluded that palea and lemma mainly develop in booting stage. During the booting stage, the palea and lemma interlock with each other to enclose the developing floral organs [24, 38].

For leaves and sheath both at booting stage, the process of photosynthesis, photorespiration, and fatty acid biosynthesis are much stronger in leaves than in sheath, which is consistent with the actual situation that leaves are responsible for photosynthesis. For comparison of endosperm with embryo, the starch and sucrose metabolism were upregulated in endosperm. The endosperm of rice is a major storage tissue for starch and proteins, which is essential for embryo growth and postembryonic development prior to the start of seedling photosynthesis [41]. The gene expression of nitrogen metabolism and fatty acid biosynthesis are downregulated in endosperm, but their fluxes are increased, which demonstrated that endosperm can accumulate more nutrients than embryo. All the above different patterns between gene expression and metabolic flux may affected by potential complex post-transcriptional modification [39, 40].

## Conclusions

This study revealed the systematic transcriptomic profile of eight *Oryza sativa* tissues in booting and mature grain stage. By further integrating transcriptome data with the rice metabolic model, we uncovered metabolic pattern shift among different tissues and stages, which can be exploited to uncover the mechanism of the complex metabolic behavior of rice and further improve production and quality of rice.

## Methods

### Tissue samples of *Oryza sativa*

The rice line of *Oryza sativa* L. ssp. japonica., cv. Zhonghua 11, were grown under natural growth conditions at an experimental field. The tissue samples of Zhonghua 11 were collected from leaves, sheath, stamen, pistil, lemma and palea of the booting stage, and from embryo, endosperm, and lemma and palea of the mature grain stage. Tissue samples were separated from the connecting tissues with a scalpel under a dissecting microscope before they were collected for RNA extraction.

### RNA isolation, library construction and sequencing

Total RNA from the eight *O. sativa* tissue samples were extracted following the manufacturer’s instructions. RNA degradation and contamination was monitored on 1% agarose gels. RNA purity was checked using the NanoPhotomete® spectrophotometer (IMPLEN, CA, US). RNA concentration was measured using Qubit® RNA Assay Kit in Qubit® 2.0 Flurometer (Life Technologies, CA, US). The RIN number was checked to determine RNA integrity by RNA Nano 6000 Assay Kit of the Agilent Bioanalyzer 2100 system (Agilent Technologies, CA, US).

A total amount of 3 μg RNA per sample was used as input material for the RNA sample preparations. Sequencing libraries were generated using NEBNext® Ultra™ RNA Library Prep Kit for Illumina® (NEB, USA) following manufacturer’s recommendations and index codes were added to attribute sequences to each sample. Briefly, mRNA was purified from total RNA using poly-T oligo-attached magnetic beads. Fragmentation was carried out using divalent cations under elevated temperature in NEBNext First Strand Synthesis Reaction Buffer (5X). First strand cDNA was synthesized using random hexamer primer and M-MuLV Reverse Transcriptase (RNase H-). Second strand cDNA synthesis was subsequently performed using DNA Polymerase I and RNase H. Remaining overhangs were converted into blunt ends via exonuclease/polymerase activities. After adenylation of 3′ ends of DNA fragments, NEBNext Adaptor with hairpin loop structure were ligated to prepare for hybridization. In order to select cDNA fragments of preferentially 150~ 200 bp in length, the library fragments were purified with AMPure XP system (Beckman Coulter, Beverly, US). Then 3 μl USER Enzyme (NEB, US) was used with size-selected, adaptor-ligated cDNA at 37 °C for 15 min followed by 5 min at 95 °C before PCR. Then PCR was performed with Phusion High-Fidelity DNA polymerase, Universal PCR primers and Index (X) Primer. At last, PCR products were purified (AMPure XP system) and library quality was assessed on the Agilent Bioanalyzer 2100 system.

The clustering of the index-coded samples was performed on a cBot Cluster Generation System using TruSeq SR Cluster Kit v3-cBot-HS (Illumia) according to the manufacturer’s instructions. After cluster generation, the library preparations were sequenced on an Illumina Hiseq 2500 platform and 125 bp paired-end reads were generated.

### Reads mapping and expression estimation

Sequencing reads were filtered according to fastq_quality_filter of fastx_toolkit-0.0.14, a command line tools for Short-Reads FASTA/FASTQ files preprocessing (−q 30). The genome and gene models of Oryza. sativa were downloaded from RAPDB (http://rapdb.dna.affrc.go.jp/download/irgsp1.html). Clean reads were aligned onto the Os-Nipponbare-Reference-IRGSP-1.0 reference genome using TopHat (version 2.0.12) with qdefault parameters [18]. Gene models were reconstructed by Cufflinks (version 2.2.1) based on the alignment results from TopHat. Fpkm (Fragment per Kilobase per Million mapped reads) used to quantify expression abundance of transcripts in each tissue [19], calculated as following:

$$ \mathrm{fpkm}=\frac{total\ exon\ Fragments}{mapped\ reads(millions)\ast exon\ length(Kb)} $$

KEGG and GO annotations were grabbed from reference gene GFF file (http://rapdb.dna.affrc.go.jp/download/irgsp1.html).

### Reconstruction of tissue-specific metabolic models in different tissues

We used the comprehensive genome-scale metabolic model of rice (iOS2164), including 2165 genes, 2444 reactions and 1999 metabolites, which is classified into 59 major metabolic sub-systems [22]. Model iOS2164 contains possible electron transfer reactions presented in mitochondria, plastids and thylakoid, as well as detailed fatty acid metabolism, intracellular lipid metabolism, and transport of metabolites.

The Integrative Metabolic Analysis Tool (iMAT) method was used to integrate transcriptome data with the rice metabolic model to reconstruct tissue-specific models. We defined “highly expressed” and “lowly expressed” genes according to their expression level, corresponding to the highest 15% genes and the lowest 15% genes respectively [16]. Next, according to the gene-protein-reaction rules and the defined gene expression states, specific activity state for each reaction was identified. This method then maximized the number of reactions whose activity is consistent with their expression state by formulating a mixed integer linear programming (MILP) problem satisfying stoichiometric and thermodynamic constraints. Finally, we got a steady-state metabolic flux distribution from different specific models. The iMAT method was conducted using the function ‘createTissueSpecificModel’ in COBRA toolbox in MATLAB, the input data for ‘createTissueSpecificModel’ including rice model, expression data, and transformation of reaction activity can be downloaded from Additional file 2.

### Total variation of fluxes across different tissues and stages

To analyze the fluctuation of each reaction flux across different tissues in different stages, we used the concept of total variation in statistics (Eq. 1).

1 $$ total\ variation={\sum}_{i=1}^k\left|{f}_i-{f}_{i-1}\right| $$

Where, f_i_ is the flux value in the i-th tissue-specific model. The greater the total variation of a reaction is, the fluctuation of this reaction is more remarkable.

### Functional enrichment analysis of up- and down-regulated genes and reactions

For the comparison of the same tissue in different stages or the different tissues in same stage, we need to determine the significantly up- and down-regulated genes and reaction fluxes. The genes with fold change of fpkm between two tissues larger than 2 or smaller than 0.5 are regarded as significantly up- and down-regulated respectively. While for metabolic level, the fluxes carried by reactions may be negative because of reversibility, therefore the simple metric like fold change is not suitable for up- and down-regulation definition. Here we calculated the difference of absolute of two flux values and divided by the range of flux variability to get the ratio of flux change, as shown in Eq. 2.

2 $$ \mathrm{ratio}=\frac{\left| flux2\right|-\left| flux1\right|}{maxflux- minflux} $$

Where the maxflux and minflux represent the maximal and minimal value of flux variability, derived by the function ‘fluxVariabilityAnalysis’ in COBRA toolbox in MATLAB. The reactions with the ratio larger than 0.5 are determined as upregulated, while those with the ratio smaller than − 0.5 are determined as downregulated. Then we conducted functional enrichment analysis using hypergeometric test in MATLAB, to find the significantly up- and down-regulated pathways between three compared pairs at the two different levels.

## Additional files

Additional file 1: The list of primary and secondary metabolic pathways in rice. (DOCX 13 kb)

Additional file 2: Input data for iMAT to construct tissue-specific models, including rice model, expression data, and transformation of reaction activity. (TIFF 3788 kb)

Additional file 3: Metabolic flux distribution of primary metabolism in different tissues at booting and mature grain stages. (ZIP 3206 kb)
