# Supplementary material for: Transcriptomic and metabolic flux analyses reveal shift of metabolic patterns during rice grain development
Source: BMC Syst Biol. 2018 Apr 24;12(Suppl 4):47. doi: 10.1186/s12918-018-0574-x (PMC5998905; doi:10.1186/s12918-018-0574-x)
Supplement: Supplementary file 1 — The list of primary and secondary metabolic pathways in rice. (DOCX 13 kb) [file 12918_2018_574_MOESM1_ESM.docx]

The list of primary and secondary metabolic pathways in rice

|  | pathway | pathway |
| --- | --- | --- |
| primary  metabolism | Glycolysis / Gluconeogenesis | Lysine degradation |
|  | Citrate cycle (TCAcycle) | Arginine and proline metabolism |
|  | Pentose phosphate pathway | Histidine metabolism |
|  | Ascorbate and aldarate metabolism | Tyrosine metabolism |
|  | Fatty acid biosynthesis | Phenylalanine metabolism |
|  | Fatty acid degradation | Tryptophan metabolism |
|  | Oxidative phosphorylation | Starch and sucrose metabolism |
|  | Photosynthesis | N-Glycan biosynthesis |
|  | Purine metabolism | Glycerolipid metabolism |
|  | Pyrimidine metabolism | Glycerophospholipid metabolism |
|  | Alanine, aspartate and glutamate metabolism | Sphingolipid metabolism |
|  | Glycine, serine and threonine metabolism | Pantothenate and CoA biosynthesis |
|  | Cysteine and methionine metabolism | Nitrogen metabolism |
|  | Valine, leucine andisoleucine degradation | Sulfur metabolism |
|  | Valine, leucine andisoleucine biosynthesis | Aminoacyl-tRNA biosynthesis |
|  | Lysine biosynthesis |  |
| Secondary  metabolism | Thiaminemetabolism | Porphyrin and chlorophyll metabolism |
|  | Riboflavinmetabolism | Terpenoid backbone biosynthesis |
|  | Vitamin B6 metabolism | Brassinosteroid biosynthesis |
|  | Nicotinate and nicotinamide metabolism | Zeatin biosynthesis |
|  | Biotin metabolism | Flavonoid biosynthesis |
