# Supplementary figures and images for: Transcriptomic and metabolic flux analyses reveal shift of metabolic patterns during rice grain development
Source: BMC Syst Biol. 2018 Apr 24;12(Suppl 4):47. doi: 10.1186/s12918-018-0574-x (PMC5998905; doi:10.1186/s12918-018-0574-x)

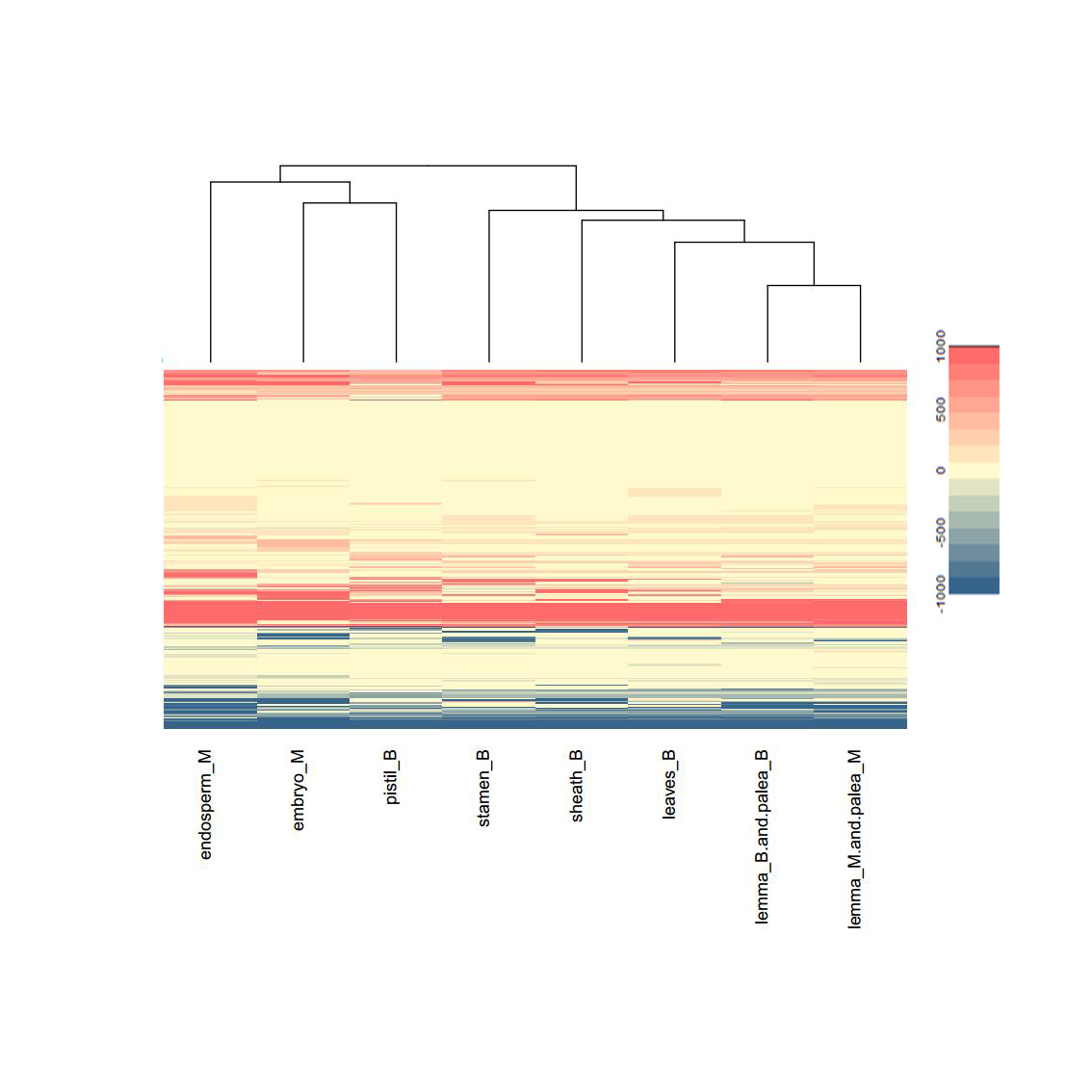

Supplement: Supplementary file 2 — Input data for iMAT to construct tissue-specific models, including rice model, expression data, and transformation of reaction activity. (TIFF 3788 kb) [file 12918_2018_574_MOESM2_ESM.tif]
